# Biologics Targeting in the Treatment of Inflammatory Bowel Disease: A Conundrum

**DOI:** 10.7759/cureus.10621

**Published:** 2020-09-23

**Authors:** Mahnoor Rehman, Ivan Cancarevic, Beshoy Iskander, Sanee Lalani, Bilal Haider Malik

**Affiliations:** 1 Internal Medicine, California Institute of Behavioral Neurosciences & Psychology, Fairfield, USA

**Keywords:** inflammatory bowel disease, inflammation, biologics, biological therapy, diarrhea

## Abstract

Inflammatory bowel disease is a chronic, gastrointestinal disorder which is classified into Crohns’ disease and ulcerative colitis. It has a strong effect on the quality of life and is characterized by chronic periods of exacerbation and remission. It has an unknown etiology but is driven due to excessive immune response in the gut wall. The triggered immune response causes overproduction of proinflammatory cytokines and adhesion molecules. Biological therapies are the monoclonal antibodies that are created in the laboratory to stop certain proteins in the body causing inflammation. These biologics have dramatically changed the therapeutic approach to inflammatory bowel disease. Biologics has three classes: anti-tumor necrosis factor (TNF), anti-integrins, and anti-interleukin (IL) 12/23. This article offers a critical evaluation of the efficacy and safety of biological agents in the management of inflammatory bowel disease. We compared different studies that were available in the PubMed database. All the biologics showed a better clinical response and mucosal healing than placebo. Infliximab has the highest efficacy, but it can make antibodies to infliximab that causes loss of response; then golimumab is effective in these patients. Certolizumab is more effective if it is used as a first-line drug. If corticosteroid and immunomodulator therapy has failed then vedolizumab is effective. As steroid therapy causes major adverse effects and involves the whole body, biological therapy should take over. Still, we need more studies to make biological therapy as a first option in the treatment of inflammatory bowel disease.

## Introduction and background

"*All diseases begin in the gut*" --Hippocrates (460-370 BC)

The estimated prevalence of inflammatory bowel disease in the USA is 1.5 million individuals and 2.2 million individuals in Europe [1]. Inflammatory bowel disease is a chronic, polygenic immune disorder of the gut. It decreases the quality of life and it can result in disability. It is a relapsing and remitting disease usually accompanied by extraintestinal manifestations for example joint, ocular, skin, liver and bile duct inflammation. Inflammatory bowel disease is divided into two types on the basis of clinical features and distinct pathology: ucerative colitis (UC) and Crohn’s’ disease (CD). Ulcerative colitis involves superficial mucous in a continuous manner. Superficial ulcers and crypt abscesses are formed by invasion of inflammatory infiltrates such as neutrophils, lymphocytes, Plasma cells and macrophages in epithelium. Ulcerative colitis starts in the rectum and involves the colon only. It does not involve small intestines whereas in Crohn’s’ disease any part of the gut from mouth to anus can be involved. In Crohn’s’ disease, inflammation extends transmurally in a discontinuous manner. In the early phase, the lymphoid aggregates give rise to aphthous ulcers and non-caseating granulomas. In the late phase, large ulcers are formed when the lymphoid aggregates extend transmurally involving submucosa and muscularis propria. These large ulcers can be the cause of fistulas and abscesses followed by strictures and fibrosis [2,3]. The symptoms are chronic diarrhea, abdominal pain, fever, weight loss, alternating flares and rectal bleeding. Anorexia and cachexia are due to increased inflammatory mediators like tumor necrosis factor (TNF) [4,5].

The etiology of inflammatory bowel disease is not clear but it is the result of a defective immune system which is a combination of genetic factors, environmental factors, intestinal flora and immune response. Environmental factors or intestinal flora triggers the immune response in the subjects who are already genetically predisposed [6,7]. Due to the induction of abnormal immune systems, there is an overproduction of proinflammatory cytokines and adhesion molecules. The activated T-cell is increased and apoptosis of T-cell is decreased [8].

**Figure 1:**
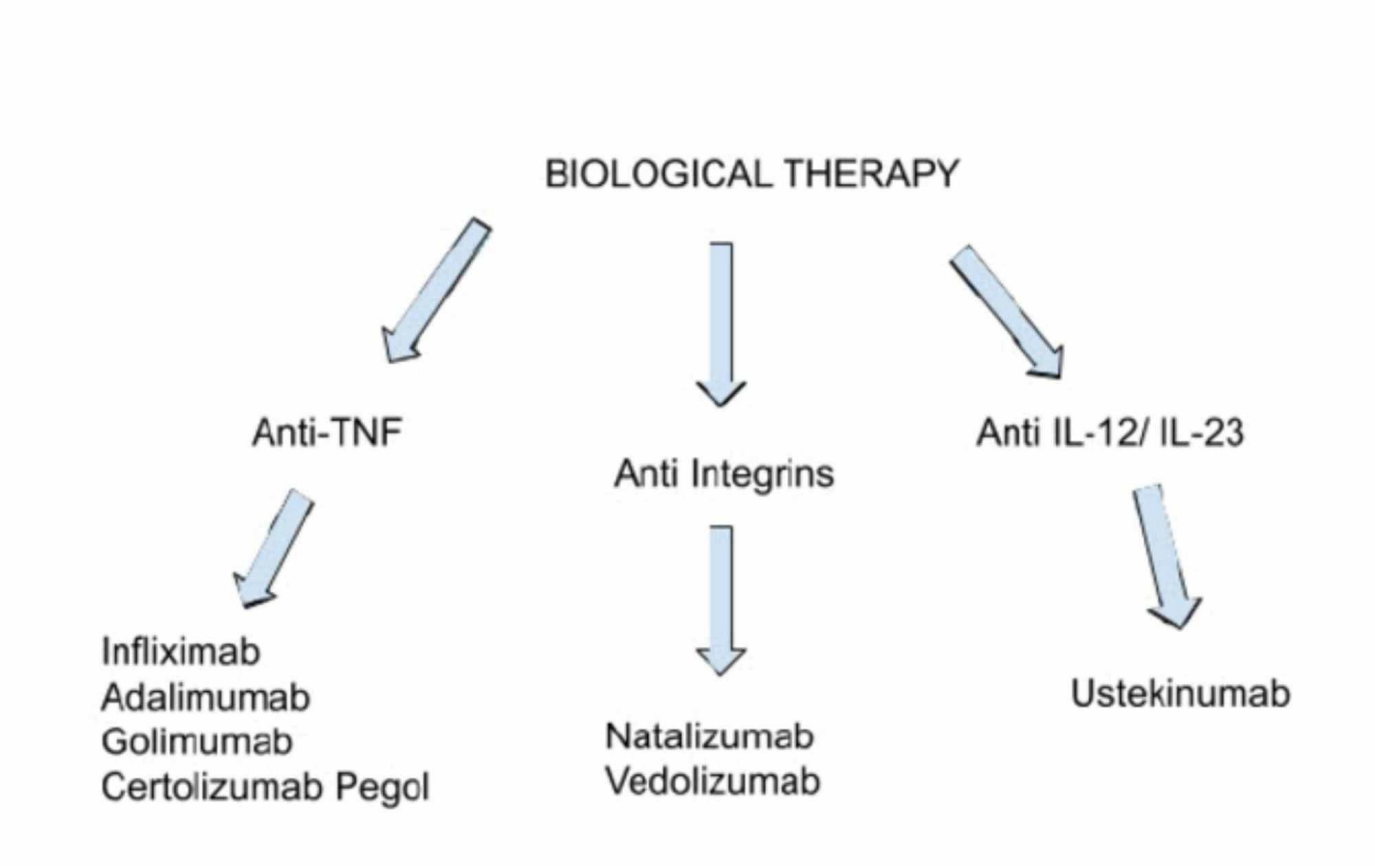
Biological therapy for IBD TNF: tumor necrosis factor; IL: interleukin

Increased cases of inflammatory bowel disease have urged the efforts to optimize medical therapy by decreasing inflammation, improving the quality of life and induction of remission of the disease without immunosuppressive drugs [9]. Biological therapies are monoclonal antibody biologics to treat inflammatory bowel disease. It has three classes -- anti-TNF, anti-integrins and anti-interleukin (IL) 12/23 (Figure 1). Anti-TNF agents are the first class that inhibit cytokine TNF-alpha, approved by FDA for the treatment of Inflammatory bowel disease [10]. Anti-TNF drugs are infliximab, adalimumab, certolizumab pegol, and golimumab.

The second class of biological therapies is anti-integrins. The inflammation is triggered by lymphocyte recruitment and migration into intestinal mucosa. Endothelial cells, mediated by integrins, endothelial adhesion molecule and chemokine receptors as intercellular adhesion molecule 1 (ICAM-1), vascular cell adhesion molecule 1 (VCAM-1), and mucosal addressin cell adhesion molecule 1 (MAdCAM-1), adhere to leukocytes [8]. Anti-integrin agents block integrins and hence interfere with the migration of leukocytes to the site of inflammation. Integrins are transmembrane receptors present on the inflammatory cells that help in cell adhesion, signalling and migration.

The third and most recent class is anti-IL 12/23 which inhibits the shared p40 subunit [10].

In this article, we reviewed the available data and compare different studies to assess the efficacy of biological therapy in inflammatory bowel disease.

## Review

We will analyze a few studies found on the PubMed database to compare the efficacy of biological therapy drugs.

Anti-tumor necrosis factor

Infliximab

Infliximab is a chimeric monoclonal antibody with high affinity to bind with alpha-TNF on macrophages and T-cells and it causes cell lysis. Hyams et al. enrolled 112 patients in a research to evaluate the efficacy of infliximab in children. Infliximab 5mg/kg was given to the patients at week zero, two, and six. The patients who responded to this drug till week 10 were divided into two equal groups randomly in order to get subsequent infusions either every eight- or twelve-week intervals. At week 10, the clinical response was 88.9% and clinical remission was 58.9%. At week 54, the clinical response of the patients receiving every eight-week infliximab was 63.5% and clinical remission was 55.8%. And the clinical response and clinical remission of the patients given every 12-week infliximab was 33.3% and 23.5%, respectively. It is observed that the clinical response and clinical remission in every 12 weeks are lower than every eight weeks. This drug is effective in children as its efficacy was 88% at week 10 [11]. Papamicheal et al. performed a retrospective study to overcome the immunogenicity of infliximab. As we know antibodies to infliximab cause immunogenicity of infliximab and subsequent treatment failure. But according to his study treatment failure is because of increased concentration of antibodies of infliximab. If there is a low concentration of antibodies of infliximab, then it does not affect the efficacy of infliximab. Then, he enrolled 22 patients on infliximab therapy and followed up with them for 17.5 months. Only 15 out of 22 patients remained on the therapy. This shows that 75% of patients have positive responses to infliximab [12]. Ben-Horin et al. performed a retrospective analysis to investigate that the infliximab therapy failure due to antibodies to infliximab can be overcome by immunomodulators and improve the clinical response of infliximab. He took five patients who have developed antibodies to infliximab and gave them immunomodulators. Two patients were given methotrexate and three were given azathioprine/6-mercaptopurine. The concentration of serum antibodies to infliximab were measured before and after the immunomodulators were given. The concentration of antibodies to infliximab was decreased after immunomodulators and clinical response was restored in all patients. This shows that the addition of immunomodulators can restore the response of infliximab in the patients [13]. In the comparison of these studies, the study of Ben-Horin et al. is the weakest as the number of patients is very small.

Adalimumab

Adalimumab is a recombinant human monoclonal antibody which binds with tumor necrosis factor-alpha and blocks the TNF receptor. It also causes lysis of cells with surface TNF and complement. A study by Colombel et al. surveyed 1,094 patients for safety and efficacy of adalimumab in moderately to severely active ulcerative colitis. He performed three placebo-controlled studies, ULTRA (Ulcerative Colitis Long-Term Remission and Maintenance in Adalimumab) 1, 2, and 3. Six hundred patients were enrolled in ULTRA 1 and 2. On follow up after four years, 199 patients remained on adalimumab therapy. Rates of remission per partial mayo score at week 208 was 24.7%. 588 patients were enrolled in ULTRA 3. On follow up after three years, 360 patients remained on adalimumab therapy. Rates of remission per partial mayo score after three years was 63.6%. Adalimumab therapy for inflammatory bowel disease is well tolerated for four years. The quality of life, remission and mucosal healing is maintained by adalimumab [14]. Paul et al. explain the efficacy and clinical response of adalimumab associated with antibodies against adalimumab (AAA) and trough levels of adalimumab (TRA). He conducted 14 systematic review studies enrolling 1,941 patients of Inflammatory bowel disease. Out of which 13 studies showed a correlation between high TRA and clinical response. However, immunosuppressive therapy does not affect the efficacy of adalimumab and TRA. But dose-escalation time was increased by combination therapy. Only one study showed no relation between high TRA and clinical response. He also conducted seven meta-analysis studies. Six studies enrolling 536 patients showed a negative correlation between AAA and clinical response. There is a higher risk in the loss of clinical response with positive AAA and if the TRA is high then the clinical response is good too [15]. Sandborn et al. conducted research to evaluate the efficacy of adalimumab and maintenance of clinical remission in moderate to severe ulcerative colitis. The study involved 494 patients. The patients were given adalimumab 160mg in the start then 80mg at week two and then 40mg every other week or placebo. The follow up was at week eight and 52. At week eight, rates of clinical remission were 16.5% on adalimumab and 9.3% on placebo. Then at week 52, the rates of clinical remission were 17.3% on adalimumab and 8.5% on placebo. Therefore, this study proves that adalimumab has better results in clinical remission than placebo [16].

In these studies, it is clear that adalimumab is well tolerated for years and its clinical response is good in patients of inflammatory bowel disease. Corticosteroid does not affect the efficacy of adalimumab. Adalimumab shows better results in clinical remission. 

In contrast to these studies, in Colombel’s study, the timing of the first dose was not the same for all patients due to the study design. So to overcome this, the patients were enrolled in ULTRA 1, 2, and 3 [14]. In Paul’s study, if the analysis was limited to adults then it was stronger and had a larger number of patients [15]. And Sandborn’s study is a subgroup analysis and the number of patients is small [16].

Golimumab

Martineau et al. conducted a retrospective study for 9.8 months to report the efficacy and safety of golimumab; the total number of patients was 115. After 3.8 months, the clinical response was 55.8%. This study shows that after the failure of infliximab and adalimumab therapy, golimumab is beneficial for the patients [17]. Gibson et al. presented a study in which the efficacy and safety of using golimumab subcutaneously were assessed in patients with mild to severe ulcerative colitis for two years of maintenance therapy. The patients treated for 52 weeks with placebo, golimumab 50mg or golimumab 100mg for every four weeks and evaluated in the 54th week were eligible. During the 104th week, almost 86% of patients were enabled to continue mild disease activity. The safety of this drug was similar to that reported in the 54th week. The use of golimumab in maintenance therapy for two years was proved beneficial without new safety signals [18]. Sandborn et al. performed double-blind trials in patients who completed induction therapy of golimumab. The patients entered maintenance therapy of 50mg of golimumab, 100 mg of golimumab and placebo every four weeks till week 52. At week 54, the clinical response was 47%, 49.7%, and 31.3% in the patients who received 50mg of golimumab, 100mg of golimumab, and placebo, respectively. The patients who received 100mg of golimumab had clinical remission and healing at week 30 and week 54 more than the patients who received placebo. This study shows the safety profile of golimumab [19]. These studies display the efficacy and safety of golimumab. This drug can be used in maintenance therapy for over two years with no new safety profile. The study of Martineau et al. is the weakest as it has a small number of populations and there is no endoscopic data present.

Certolizumab Pegol

Certolizumab pegol is a pegylated humanized fab fragment. It has a high affinity for alpha-tumor necrosis factor but it does not cause T-cell or monocyte apoptosis. Schreiber et al. performed a randomized, double-blind, placebo-control trial on 668 patients to evaluate the efficacy of certolizumab. The patients were given 400mg of certolizumab subcutaneously at week zero, two, and four. At week six, 428 out of 668 patients responded to the induction therapy and entered the maintenance therapy. Three patients were excluded according to the exclusion criteria. In maintenance therapy, 210 patients were given placebo and 215 patients were given certolizumab 400mg every four weeks. The clinical remission in the patients who received placebo was 51.4% and 69.9% in the patients who received certolizumab. This study proposed that the clinical remission and the medical therapy response in patients who were given maintenance therapy were better than those who received placebo [20]. In a study, the efficacy and safety of certolizumab were presented by Moon et al. He enrolled 358 patients who had already failed biological therapies, 78.8% infliximab, 63.7% adalimumab and 2.8% natalizumab. In 112 patients, certolizumab was the second biological agent and in 189 patients it was the third biological agent. The results showed the clinical benefits of certolizumab in patients who have already failed the biological therapies. Many other early reports agree with this result. This drug can be more effective if it is used as a first-line or second-line drug [21]. Stein et al. conducted a retrospective chart review for certolizumab pegol dosage and clinical response. He enrolled 87 patients, mostly failed biological therapies. The clinical response was positive in only 27 out of 87 patients that is 31%. Then 31 patients were re-induced and only five patients showed positive clinical response, that is, 16.1%. This study shows that certolizumab gave less beneficial results as a second-line or third-line agent, and this may be due to loss of response mechanisms or immunogenicity. This drug can be more effective if it is used as a first-line agent [22].

In the comparison of the studies, the Stein et al. study is weak as it is a small-sized retrospective study of 100 patients. The results are not reliable as this study is based on different populations and referral-based academics.

Anti-integrin

Natalizumab

Natalizumab is a human monoclonal antibody against cell adhesion molecule α4-integrin. The drug is commonly used to treat chronic inflammatory conditions such as inflammatory bowel disease. The drug acts by stopping the migration of inflammatory cells across the cell layers. A study was conducted on 248 patients to assess the efficacy of natalizumab. The patients were divided into four groups. The first group received two placebo doses, the second group was given one dose of 3mg of natalizumab and one placebo, the third group received two doses of 3mg of natalizumab and the fourth group received two doses of 6mg of natalizumab. The doses were given four weeks apart. Remission rate was increased in the groups who were given two infusions of natalizumab. The rate of remission was 44% and the rate of response was 71%. This study shows the short-term efficacy and safety of natalizumab in patients with Crohn’s’ disease [23]. In another study, the efficacy of the drug was tested on 10 patients who were given an infusion of 3mg of natalizumab. At week two, five out of 10 patients showed a positive clinical response. At week four, one more patient showed a positive clinical response. So the conclusion made was that this drug is safe, well-tolerated and improves the quality of life [24]. Sandborn et al. conducted two controlled trials to assess the induction and maintenance therapy of natalizumab. They enrolled 905 patients who received 300mg of natalizumab or placebo. The Crohn's activity was decreased by 70 points in week 10. The drug did not show a good clinical response. The results were almost the same as the patients who were given a placebo. 339 patients out of 905, who responded to natalizumab, were given natalizumab every four weeks through week 56. The response was positive in the patients who continued natalizumab for a long time [25].

The second study of natalizumab, which was published in 2002, is the weakest as the number of patients is only 10.

Vedolizumab

Vedolizumab is a monoclonal antibody used to treat cases of refractory IBD. Patients of ulcerative colitis or Crohn’s disease for a long time lacked any treatment for situations in which corticosteroids or older immune modulators have failed. With the introduction of agents such as vedolizumab, it has become possible to treat these cases. With older agents such as TNF antagonists, maintaining a state of remission is difficult with patients having decreasing response rates when multiple TNF antagonists are used [26]. Vedolizumab is an integrin inhibitor; specifically, an a4b7 integrin inhibitor, making it a gut selective anti-inflammatory agent [27]. This specificity of the drug makes it very useful in the case of inflammatory bowel disease. However, with the introduction of this new class of medications, the question of efficacy and its ability to treat patients in actual clinical practice has been raised [27]. According to three separate research studies performed to test the efficacy of vedolizumab, this new drug is performing well. In one cohort study of vedolizumab consisting of 294 patients with inflammatory bowel disease, one-third of the patients were in steroid-free clinical remission at 14-weeks of therapy [28]. In another randomized controlled study, it was found that at 10 weeks of therapy 26.6% of the study population was in remission in comparison to the 12.1% in the placebo group [26]. In a third cohort study with a group of 172 patients found that the remission rate of the Vedolizumab group as compared to placebo was 48.9 % and 23.9% respectively for Crohn’s and 53.5% and 29.5% respectively for UC at 14th week [27]. It can easily be seen from the results of these studies that vedolizumab therapy has an effective response on remission past 10 weeks of therapy. However; it is difficult to assess how well the therapy will work for patients with different severities of disease. Since the studies had not stratified groups based on disease severity, rather had only included patients who were above certain severity levels. All in all, it can be concluded that vedolizumab is effective in patients with refractory IBD and it can be used to push patients into disease remission.

Anti-IL 12/23

Ustekinumab

Ustekinumab is a monoclonal antibody of p40 subunit of IL 12/23. Wils et al. performed a retrospective observational study on 122 patients of active Crohns’ disease, who received a subcutaneous injection of ustekinumab. These patients were followed up after three months. Out of which 79 patients showed a positive clinical response and 43 patients did not respond to this drug. It was noted that this drug is beneficial in reducing the symptoms and treating active Crohn’s disease [29]. Sandborn et al. reported that treatment response of ustekinumab in Crohn’s’ disease is better than placebo. They enrolled patients in two trials, 741 patients in one trial and 628 patients in another. They induced the patients of each trial with ustekinumab of 130mg, ustekinumab of 6mg/kg, or placebo. After six weeks, they assessed the patients and came with a result that the rate of response of ustekinumab is better than placebo. Then they gave a maintenance dose of 90mg, subcutaneously every eight weeks or every 12 weeks to 397 patients. At week 44, patients taking ustekinumab had 53.1% remission rate and 48.8% for the patients on placebo, and they observed that the remission rates were higher in the patients taking ustekinumab therapy than placebo [30]. The efficacy and safety monitoring of this drug was continued by Sandborn et al. for the second year. The patients on placebo were not included; only patients on ustekinumab therapy were enrolled. Out of 718 patients, only 621 patients completed week 92. In some patients, the dose was adjusted from every eight weeks ustekinumab injection to every 12 weeks ustekinumab injection till week 44. The dose was maintained afterwards till week 92. The efficacy of randomized patients on week 92 was 72.6%, 74.4%, and 53.3% for the patients on ustekinumab 90mg every 12 weeks, ustekinumab 90mg every eight weeks and the patients with dose adjustments. The efficacy results were the same for ustekinumab every 12 and eight weeks but lower in patients with dose adjustments. The safety events were the same for the patients on ustekinumab and placebo [31]. From these studies, it can be said that this drug is safe and has a good clinical response.

These studies show the clinical response and efficacy in patients suffering exclusively from Crohn's disease, and not ulcerative colitis. In comparison to Sandborn's study, Wils's study contains a smaller sample size, making it the weaker study.

The efficacy of different biological therapies in IBD is summarized in Table 1. 

**Table 1:** Summary of efficacy of biological therapy for IBD IBD: irritable bowel disease

Drugs	Reference (year)	Study	Study population	Study Protocol	Results
Infliximab	Hyams et al. (2006) [11]	Explains efficacy of infliximab	N=112	5mg/kg IV every 8 weeks after induction until week 54	Clinical response and clinical remission at week 54 were 63.5% and 55.8% (infusion every 8 weeks)
				5mg/kg IV every 12 weeks after induction until week 54	Clinical response and clinical remission at week 54 were 33.3% and 23.5% (infusion every 12 weeks)
	Papamicheal et al. (2018) [12]	Explains efficacy of infliximab by increase in antibodies to Infliximab	N=22	Infliximab and antibodies were administered for 17.5 months	15 patients showed positive result
	Ben-Horin et al. (2012) [13]	Explains infliximab failure due to antibodies to infliximab	N=5	Administered immunomodulator (methotrexate to 2 patients and azathioprine/6-mercaptopurine to three patients)	Clinical response was restored by decrease in antibodies to infliximab
Adalimumab	Colombel et al. (2014) [14]	Explains efficacy of adalimumab	N=1094	600 patients in ULTRA 1 and 2 for four years	Rate of remission at week 208 was 24.7%
				588 patients in ULTRA 3 for 3 years	Rate of remission after 5 years was 63.6%
	Paul et al. (2014) [15]	Explains efficacy and clinical response of AAA	N=1941	Conducted 14 systematic review studies	13 studies show correlation between high TRA and clinical response
			N=536	Conducted seven meta-analysis studies	six studies showed negative correlation between AAA and clinical response
	Sandborn et al. (2011) [16]	Explains efficacy of adalimumab	N=494	160mg of adalimumab was administered, then 80mg at week two and 40mg every other week or placebo until week 52	At week eight, clinical remission on adalimumab was 16.5% and 9.3% on placebo
					At week 52, clinical remission on adalimumab was 17.3% and 8.5% on placebo
Golimumab	Martineau et al. (2017) [17]	Reported efficacy and safety of golimumab	N=15	Golimumab was given for 9.8 months	Clinical response was 55.8% after 3.8 months
	Gibson et al. (2016) [18]	Efficacy and safety of golimumab for 2 years of maintenance therapy	N=1228	Administered placebo, golimumab 50mg and golimumab 100mg every four weeks till week 52	At week 104, 86% patients were enabled to continue mild disease activity
	Sandborn et al. (2013) [19]	Efficacy and safety of golimumab	N=1064	Administered placebo, golimumab 50mg and golimumab 100mg every four weeks till week 52	At week 54, clinical response were 47%, 49.7% and 31.3% in patients who received 50mg of golimumab, 100mg of golimumab and placebo
Certolizumab Pegol	Schreiber et al. (2007) [20]	Evaluation of efficacy of certolizumab	N=668	400mg subcutaneously at week 0,2 and four	At week 6, 428/668 patients responded to induction therapy and entered maintenance therapy
				Placebo was given to 210 patients	Clinical remission in patients receiving placebo was 51.4%
				400mg every four weeks certolizumab was given to 215 patients	Clinical remission in patients receiving certolizumab was 69.9%
	Moon et al. (2015) [21]	Efficacy of certolizumab	N=358	Certolizumab was administered in patients who have already failed biological therapies	In 112 patients, certolizumab was the second biological agent
					In 189 patients, certolizumab was the third biological agent
	Adam et al. (2014) [22]	Efficacy of certolizumab	N=87	Certolizumab was administered in patients who have already failed biological therapies	31% of patients gave a positive clinical response
				31 patients were re-induced	Only 5 patients (61.1%) showed a positive response
Natalizumab	Ghosh et al. (2003) [23]	Evaluation of efficacy of natalizumab	N=248	Patients were divided into four groups	Rate of remission was 44% and the rate of response was 71%
				1st group was given two doses of placebo	
				2nd group was given one dose of Natalizumab and one dose of placebo	
				3rd group was given two doses of 3mg of natalizumab	
				4th group was given two doses of 6mg of natalizumab	
	Gordon et al. (2002) [24]	Efficacy of natalizumab	N=10	Infusion of 3mg of natalizumab	At week two, 5/10 patients showed positive clinical response
					At week 4, one more patient showed positive clinical response
	Sandborn et al. (2005) [25]	Access induction and maintenance therapy of natalizumab	N=905	300mg of natalizumab or placebo	Results were almost the same as placebo
				339 patients who responded to natalizumab were given natalizumab every four weeks till week 56	Result was positive in patients who used natalizumab for a long time
Vedolizumab	Bruce et al. (2014 ) [26]	Evaluation of efficacy of vedolizumab	N=294	Infusion of vedolizumab for 14 weeks	1/3rd patients were in steroid-free clinical remission
	Shelton et al. (2015 ) [27]	Efficacy of vedolizumab	N=315	Infusion of vedolizumab for 10 weeks	26.6% of the study population was in remission
	Amiot et al. (2016 ) [28]	Efficacy of vedolizumab	N=172	Infusion of vedolizumab for 14 weeks	Clinical remission of patients on vedolizumab was 48.9% in Crohn’s disease
					Clinical remission of patients on placebo was 23.9% in Crohn’s disease
					Clinical remission of patients on vedolizumab was 53.5% in Ulcerative colitis
					Clinical remission of patients on placebo was 29.5% in Ulcerative colitis
Ustekinumab	Wils et al. (2015 ) [29]	Efficacy of Ustekinumab	N=122	Subcutaneous injection of ustekinumab and followed up after three months	79 patients showed a positive response
					43 patients did not respond to therapy
	Feagan et al. (2016 ) [30]	Efficacy of Ustekinumab	N=1361	He performed two trials	Rate of response of ustekinumab is better than placebo
				130mg ustekinumab, 6mg/kg ustekinumab and placebo was given to patients	At week 44, clinical remission for patients on ustekinumab was 53.1%
				Then gave maintenance dose of 90mg subcutaneously every eight weeks or every 12 weeks to 397 patients	At week 44, clinical remission for patients on placebo was 48.8%
	Sandborn et al. (2018 ) [31]	Efficacy and safety of ustekinumab	N=718	Ustekinumab 90mg every 12 weeks, ustekinumab 90mg every eight weeks was given and patients with dose adjustments till week 92	At week 92, efficacy of randomized patients was 72.6%, 74.4% and 53.3% for patients on ustekinumab 90mg every 12 weeks, ustekinumab 90mg every eight week and patients with dose adjustments

## Conclusions

In conclusion, this review article summarizes different studies to show the efficacy and safety of biological therapy in inflammatory bowel disease. Over the last several years, biological therapy is used for the treatment of inflammatory bowel disease. Biologics are the antibodies that are created in laboratories to stop certain proteins in the body from causing inflammation. All the biologics show positive clinical responses and safety. The rate of clinical remission and mucosal healing in therapy with infliximab, adalimumab, golimumab, certolizumab, vedolizumab, natalizumab and ustekinumab has shown better results than placebo. Infliximab has the highest efficacy, nearly three-fourths of patients show positive responses. Infliximab and adalimumab lose the clinical response due to antibodies generated against them; golimumab is effective in such patients. Adding immunomodulators with infliximab helps to overcome the loss of response of infliximab by the antibodies produced against it. Certolizumab is more effective if it is used as a first-line agent. Natalizumab does not give a positive clinical response if introduced for a short term. The positive results are shown when it is continued for a long term. To treat the cases in which corticosteroids and immunomodulators have failed, vedolizumab is effective. It is important to know the efficacy of biologics as they have a positive impact on management of inflammatory bowel disease. They can overcome the use of corticosteroids that affects the whole body and causes major adverse effects, as well as, they are very selective in their mechanism of action. Much about mucosal immunity is still unclear in current literature. Mucosal healing is the target in the treatment of inflammatory bowel disease and biologics show good response to the mucosal healing. The biologics used in inflammatory bowel disease are giving good results, but we need more clinical studies to make biological therapy as the first-line agents for the treatment of IBDs. 
